# Beyond Prosthetic Valve Infection: Anchoring Bias Delaying the Diagnosis of Synovial Sarcoma

**DOI:** 10.7759/cureus.115343

**Published:** 2026-08-28

**Authors:** Sai Deepika Gudapati, Kaitlin Liroff, Qazi Azher

**Affiliations:** 1 Internal Medicine, Hurley Medical Center, Flint, USA; 2 Infectious Diseases, Hurley Medical Center, Flint, USA; 3 Pathology and Laboratory Medicine, Hurley Medical Center, Flint, USA

**Keywords:** anchoring bias, diagnostic delay, fever of unknown origin, neoplastic fever, synovial sarcoma

## Abstract

Synovial sarcoma is a rare soft tissue malignancy typically presenting as a deep-seated mass in younger individuals, with atypical presentations in older adults contributing to delayed diagnosis. We report a 68-year-old woman who developed persistent intermittent constitutional symptoms, prompting evaluation for fever of unknown origin (FUO) following surgical aortic valve replacement (SAVR), initially raising concern for prosthetic valve infection. Despite repeated negative blood cultures, an unremarkable echocardiogram, and a lack of objective evidence of infection, the diagnostic focus remained anchored in prosthesis-related etiologies, reinforced by coincidental clinical events and concern for hypersensitivity. Subsequent physical examination revealed a subtle deep-seated left thigh mass. Imaging demonstrated a heterogeneous lesion measuring approximately 7-8 cm with internal vascularity, and biopsy confirmed monophasic synovial sarcoma. This case highlights anchoring bias as a major contributor to diagnostic delay, with neoplastic fever, likely cytokine-mediated, mimicking chronic infection. It underscores the importance of maintaining a broad differential diagnosis in FUO and emphasizes the value of careful serial physical examination in identifying occult malignancy.

## Introduction

Synovial sarcoma is a rare mesenchymal malignancy accounting for approximately 5-10% of all soft tissue sarcomas, with an estimated annual incidence of one to three cases per million individuals [1]. It most commonly affects adolescents and young adults between the ages of 15 and 40 years and typically arises in the deep soft tissues of the extremities, particularly in the periarticular regions of the lower limbs [2,3]. Presentation in older adults is uncommon and may contribute to delayed recognition, especially when clinical features are atypical or nonspecific [2,3]. In such cases, the diagnosis may be further obscured by competing clinical considerations and cognitive biases that reinforce more common or seemingly plausible etiologies. Here, we present a patient who initially presented with fever of unknown origin (FOU) and was ultimately diagnosed with synovial sarcoma.

## Case presentation

A 68-year-old woman with a history of severe aortic stenosis status post-surgical aortic valve replacement (SAVR), type 2 diabetes mellitus, hypertension, morbid obesity (body mass index (BMI): 51.35 kg/m²), gastroesophageal reflux disease, esophageal strictures, hiatal hernia, chronic urticaria, and presumed obstructive sleep apnea presented with persistent intermittent constitutional symptoms following valve replacement.

She underwent SAVR in August 2020 after progressive exertional symptoms and multidisciplinary evaluation, without immediate postoperative complications. Shortly thereafter, she developed recurrent episodes of subjective low-grade fever (≤99°F), diaphoresis, fatigue, intermittent nausea, and diarrhea occurring approximately every 2-3 weeks. These symptoms persisted for several months and prompted repeated outpatient and inpatient evaluations. Given the temporal association with recent prosthetic valve implantation, prosthetic valve infection became the leading diagnostic consideration early in her clinical course.

This diagnostic impression was reinforced by several coinciding clinical events. One febrile episode followed a dental procedure despite prophylactic clindamycin, and another occurred after a left first toe infection requiring antibiotic therapy including linezolid. Persistent concern for occult infection prompted repeated blood cultures and cardiac evaluations. Transthoracic and transesophageal echocardiography demonstrated no valvular vegetations or evidence of infective endocarditis, and serial blood cultures remained negative. Computed tomography of the abdomen and pelvis performed to evaluate for occult infectious complications was unrevealing. Despite the absence of objective evidence of infection, the patient received multiple empiric antimicrobial therapies during her clinical course.

Complicating the evaluation further was the patient’s reported history of nickel allergy, raising concern for hypersensitivity to implanted prosthetic material following SAVR, despite previously negative allergy testing. Nevertheless, both the patient and treating physicians remained concerned that implanted surgical material might be contributing to her persistent symptoms, reinforcing diagnostic focus on valve-related etiologies.

Despite repeated evaluations by multiple physicians - including inpatient teams, primary care providers, cardiologists, and infectious disease specialists - the underlying etiology remained elusive. The patient herself had not identified any concerning soft tissue abnormality. During infectious disease evaluation in August 2021 for persistent low-grade fevers, physical examination revealed a subtle, deep-seated, non-tender soft tissue mass involving the left thigh. The lesion lacked overlying erythema, warmth, or fluctuance and was not suggestive of abscess or cellulitis. Given concern for an infiltrative process, further imaging was pursued.

**Figure 1:**
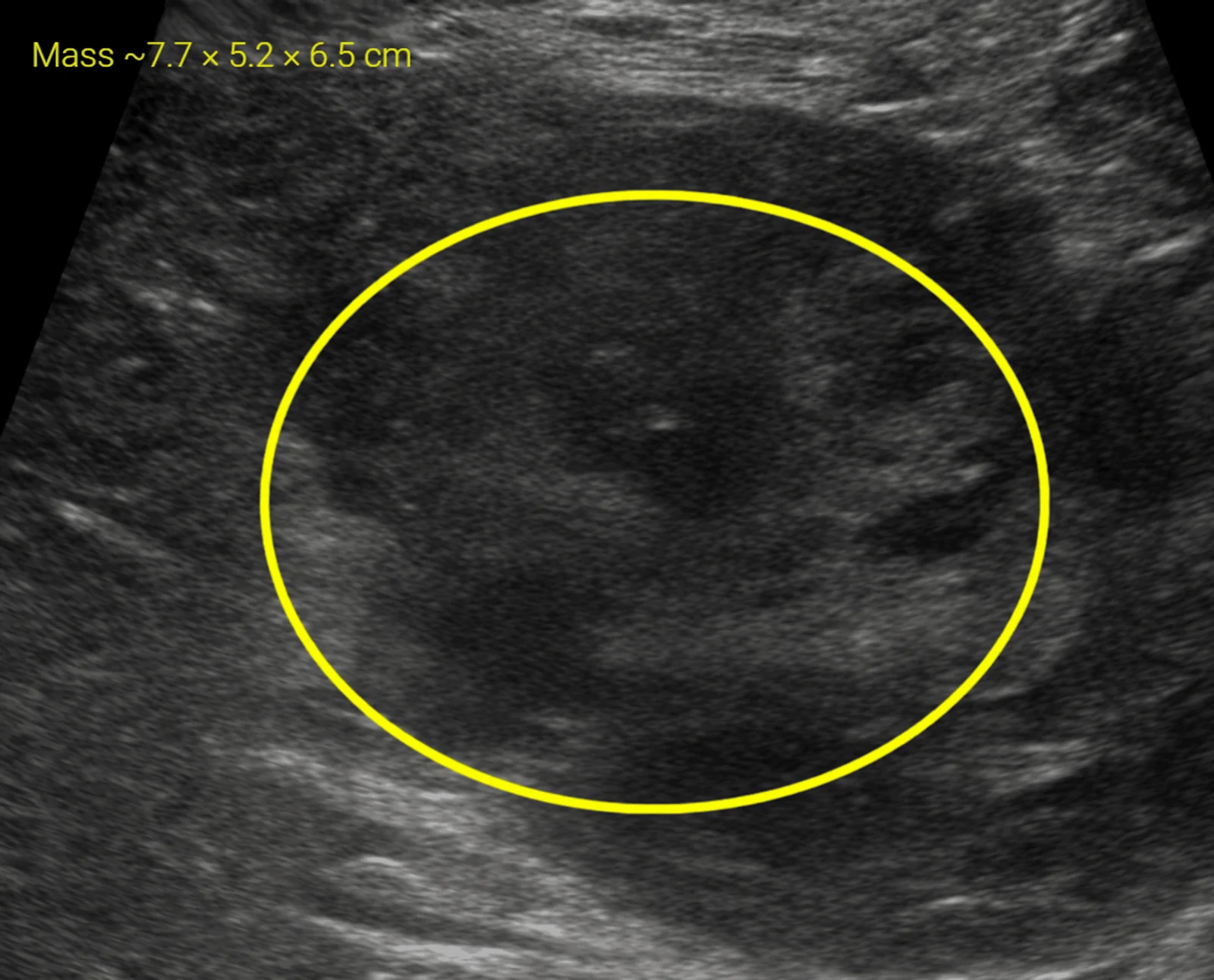
Grayscale ultrasound of the left thigh showing a large, heterogeneous, predominantly hypoechoic lesion (yellow circle).

**Figure 2:**
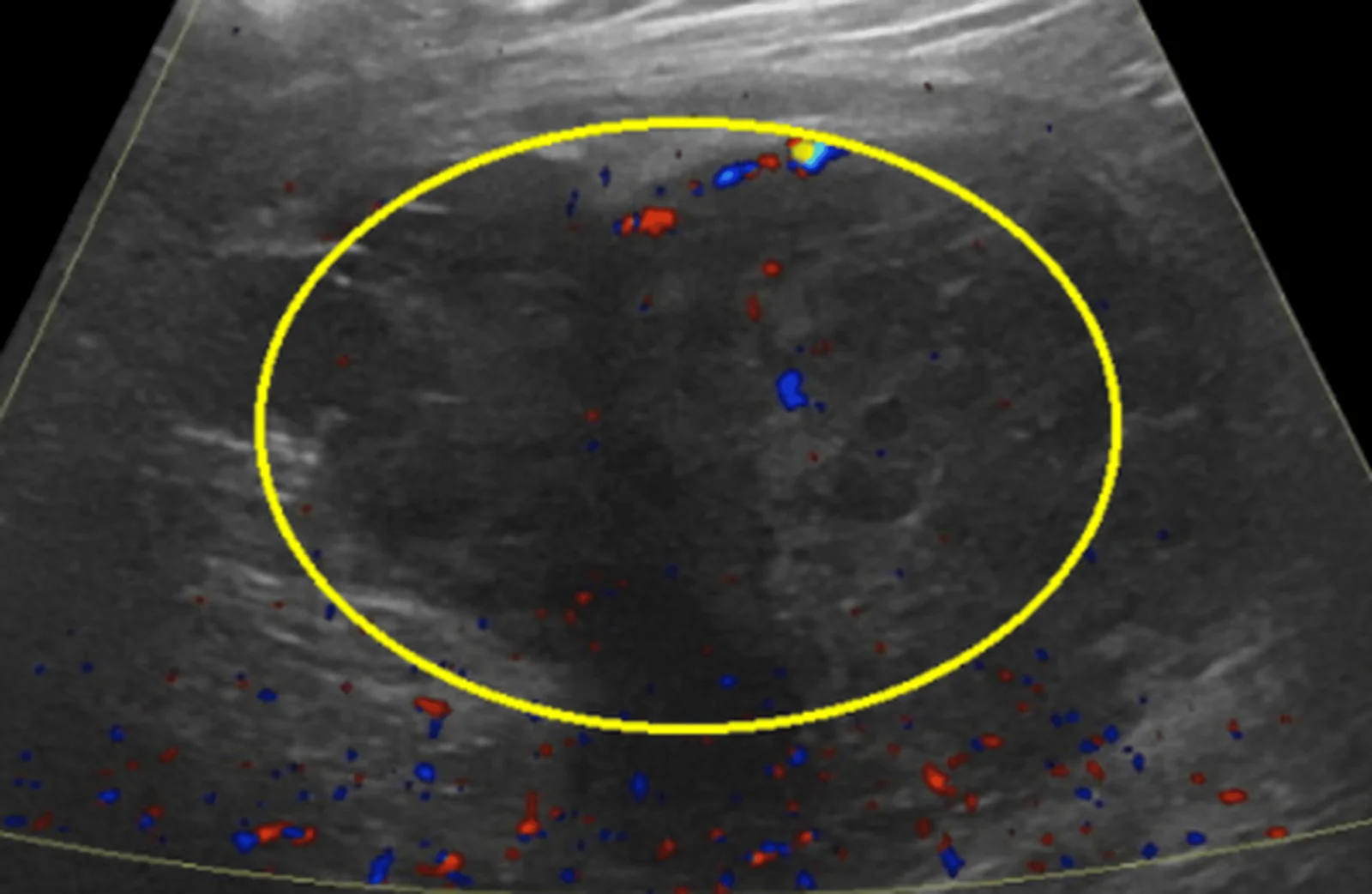
Color Doppler ultrasound demonstrating internal vascularity (yellow circle) within the lesion, supporting suspicion for a neoplastic process.

**Figure 3:**
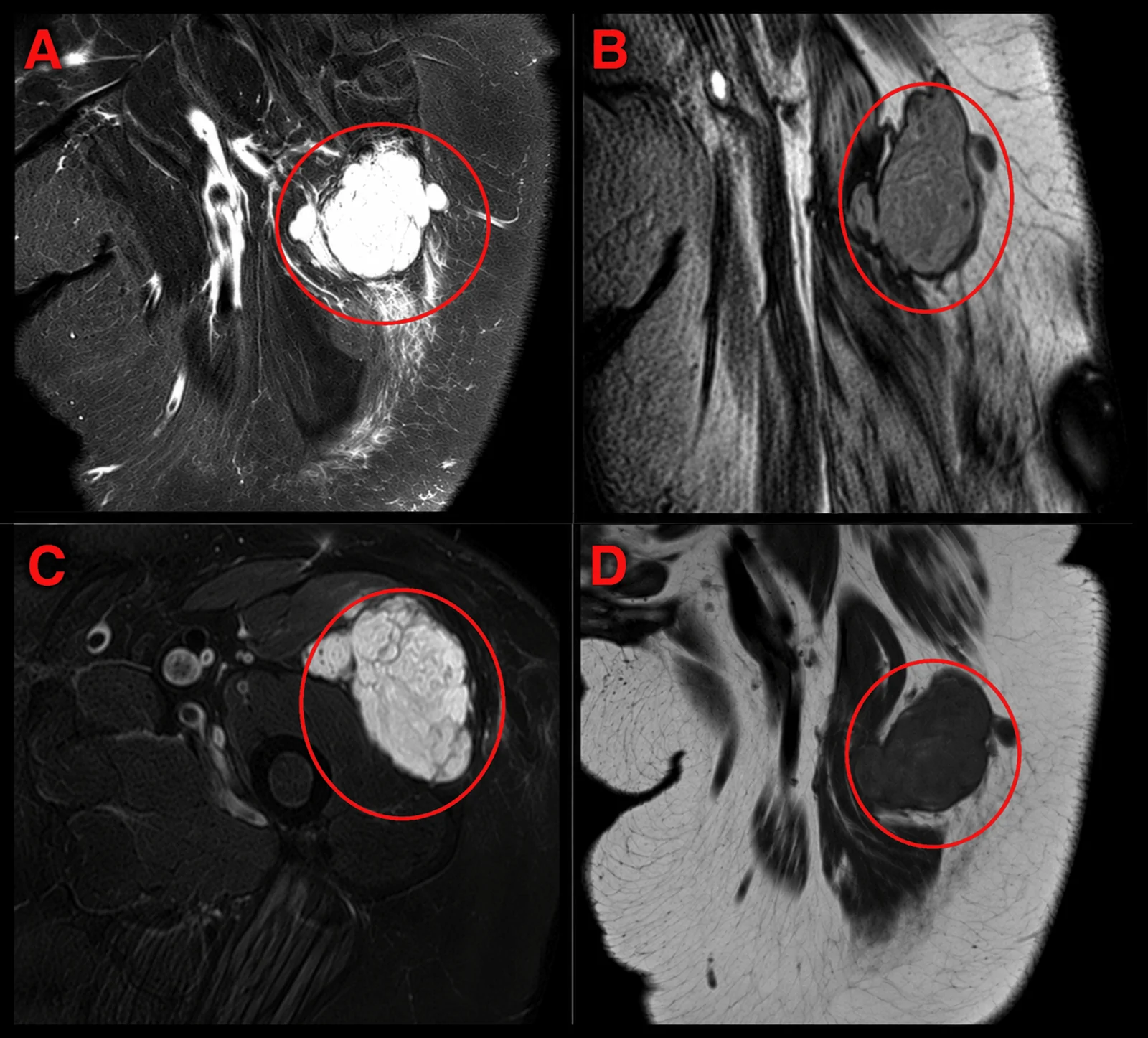
(A) Coronal T1-weighted post-contrast showing a well-circumscribed lesion (red circle) with heterogeneous enhancement. (B) Sagittal view demonstrating an ovoid, relatively homogeneous mass (red circle). (C) Axial fluid-sensitive sequence revealing a heterogeneous, hyperintense lesion with internal complexity and surrounding soft tissue edema (red circle). (D) Coronal T1-weighted pre-contrast image confirming the intramuscular location and well-defined margins of the lesion (red circle).

Ultrasound of the left thigh performed in October 2021 demonstrated a heterogeneous subcutaneous soft tissue mass with internal vascularity measuring approximately 7.7 × 5.2 × 6.5 cm, raising suspicion for a neoplastic process (Figures 1-2). Subsequent magnetic resonance imaging of the left thigh with and without contrast revealed an anterior compartment heterogeneous mass measuring approximately 6.2 × 8.1 × 6.2 cm interposed between vastus intermedius and lateralis, demonstrating substantial contrast enhancement and features suggestive of sarcoma (Figure 3). The lesion was not clearly confined to the muscle, further raising concern for an aggressive soft tissue neoplasm.

**Figure 4:**
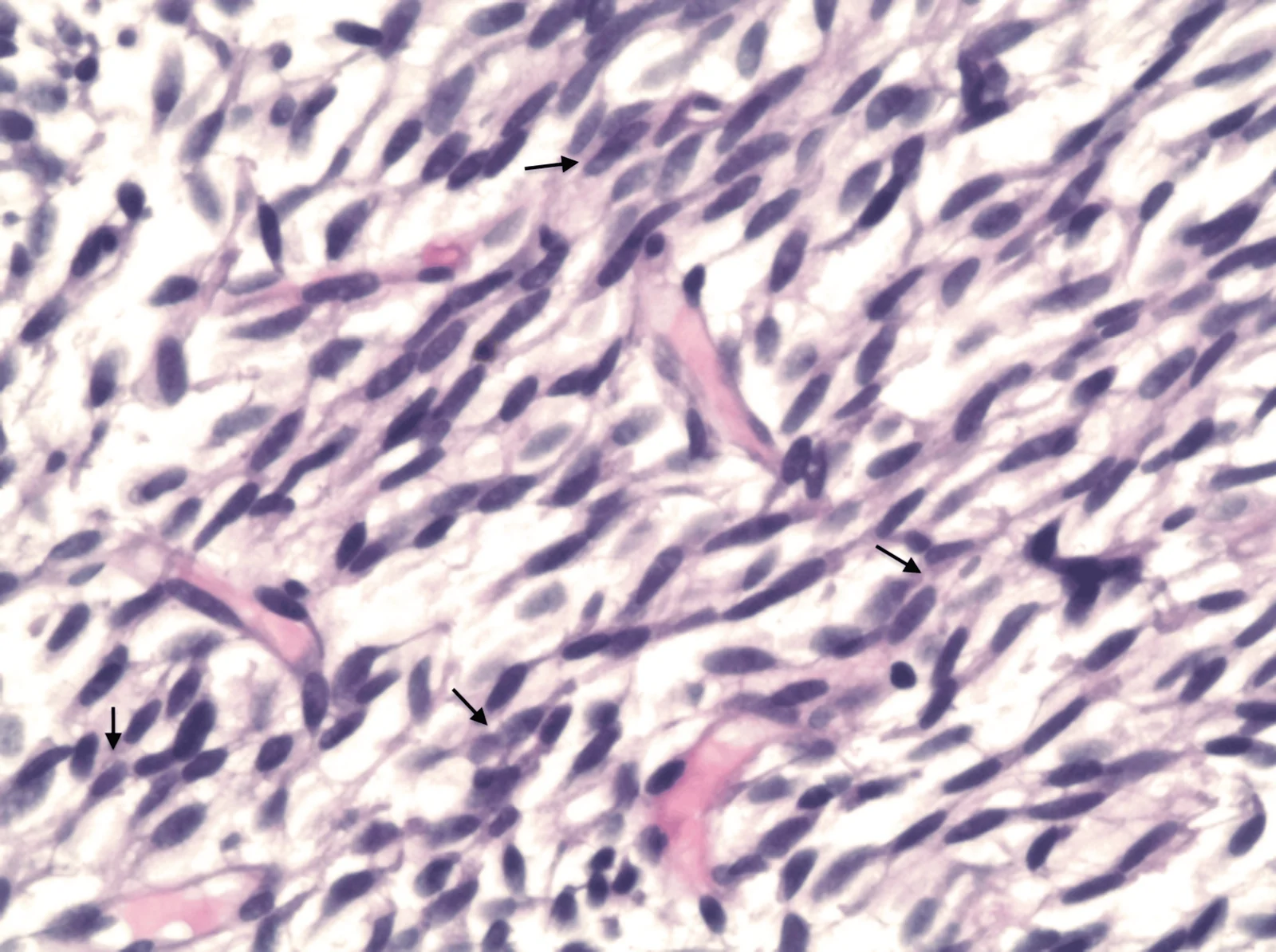
Histopathological examination (400X) of the left thigh mass showing a densely cellular spindle cell neoplasm arranged in intersecting fascicles (as indicated by arrows). Tumor cells exhibit elongated nuclei with mild-to-moderate atypia and hyperchromasia, with intervening areas of myxoid stroma, consistent with monophasic synovial sarcoma.

An ultrasound-guided core biopsy of the lesion was performed. Histopathologic examination demonstrated a cellular malignant spindle cell proliferation arranged in vague fascicles with associated areas of myxoid stroma (Figure 4). The tumor cells exhibited moderate cytologic atypia and hyperchromatic chromatin. Immunohistochemical analysis revealed that the lesional cells were strongly positive for SS18-SSX, with focal positivity for cytokeratin, and demonstrated expression of desmin and H3K27me3. The tumor was negative for STAT6 and SOX10. These morphologic and immunophenotypic features were diagnostic of monophasic synovial sarcoma.

The patient was referred to a tertiary sarcoma center, where multidisciplinary oncologic evaluation recommended neoadjuvant radiation therapy followed by surgical resection for local disease control.

## Discussion

Synovial sarcoma is a rare mesenchymal malignancy accounting for approximately 5-10% of all soft tissue sarcomas, with an estimated annual incidence of one to three cases per million individuals [1]. It most commonly affects adolescents and young adults between the ages of 15 and 40 years and typically arises in the deep soft tissues of the extremities, particularly in the periarticular regions of the lower limbs [2,3]. Presentation in older adults, as observed in this case, is uncommon and may contribute to delayed recognition.

Clinically, synovial sarcoma often presents as a slowly enlarging, deep-seated soft tissue mass that may remain painless for an extended period [4]. Constitutional symptoms such as fever, diaphoresis, malaise, and weight loss are atypical initial manifestations and may obscure the underlying diagnosis [5]. In the present case, the patient’s predominant presentation of constitutional symptoms over several months following SAVR created a compelling clinical narrative favoring prosthetic valve infection.

This case exemplifies anchoring bias, a cognitive error in which clinicians rely heavily on an initial diagnostic impression despite evolving or conflicting evidence [6]. The presumption of prosthetic valve infection became reinforced over time, even in the absence of objective findings. Intermittent clinical events, including a dental procedure and a toe infection, further strengthened suspicion for occult infective endocarditis despite repeatedly negative blood cultures and unremarkable echocardiographic studies. Additionally, concern for hypersensitivity to prosthetic material due to a reported nickel allergy further directed attention toward valve-related etiologies. Collectively, these factors illustrate how coincidental or weakly associated clinical findings can sustain diagnostic momentum toward an incorrect working diagnosis.

An important clinical lesson highlighted by this case is the subtle presentation of the underlying malignancy. The thigh lesion was deep-seated, non-tender, and lacked overlying inflammatory changes. The patient had not appreciated the mass, and it remained undetected during multiple previous evaluations. Soft tissue sarcomas frequently evade early recognition due to their indolent growth patterns and absence of prominent localizing symptoms [7]. In this instance, careful repeat physical examination proved more diagnostically valuable than extensive infectious investigations, underscoring the importance of thorough and serial clinical examination in patients with FUO. 

The pathophysiology of neoplastic fever is thought to be driven by inflammatory cytokine signaling. Elevated levels of pro-inflammatory mediators such as interleukin (IL)-1), IL-6, tumor necrosis factor-alpha (TNF-α), and interferon-gamma have been implicated [8,9]. Among these, IL-6 plays a central role in both tumor biology and systemic inflammatory response in soft tissue sarcomas. Increased IL-6 expression has been associated with constitutional symptoms, elevated inflammatory markers, tumor progression, and adverse outcomes [10,11]. These cytokine-mediated mechanisms may result in persistent fevers that closely mimic chronic infection, particularly in patients with plausible infectious risk factors, as demonstrated in this case.

Malignancy remains a well-recognized but often delayed cause of FUO [12]. While hematologic malignancies are more commonly implicated, solid tumors such as soft tissue sarcomas represent an uncommon but important diagnostic consideration [13]. This case reinforces the need to maintain a broad differential diagnosis in patients with persistent FUO despite an initially convincing alternative explanation and highlights how anchoring bias can delay recognition of occult malignancy.

## Conclusions

Synovial sarcoma, although rare, may present atypically in older adults with constitutional symptoms rather than a palpable mass and should remain in the differential diagnosis of FUO when infectious investigations are unrevealing. Maintaining diagnostic flexibility and performing careful serial physical examinations are essential to identifying uncommon but clinically significant causes of persistent constitutional symptoms.

## References

[1] Sultan I, Rodriguez-Galindo C, Saab R, Yasir S, Casanova M, Ferrari A (2009). Comparing children and adults with synovial sarcoma in the Surveillance, Epidemiology, and End Results program, 1983 to 2005: an analysis of 1268 patients. Cancer.

[2] Thway K, Fisher C (2014). Synovial sarcoma: defining features and diagnostic evolution. Ann Diagn Pathol.

[3] Trama A, Badalamenti G, Baldi GG (2019). Soft tissue sarcoma in Italy: from epidemiological data to clinical networking to improve patient care and outcomes. Cancer Epidemiol.

[4] Clark MA, Fisher C, Judson I, Thomas JM (2005). Soft-tissue sarcomas in adults. N Engl J Med.

[5] Nakamura T, Matsumine A, Matsubara T, Asanuma K, Sudo A (2016). Neoplastic fever in patients with bone and soft tissue sarcoma. Mol Clin Oncol.

[6] Croskerry P (2003). The importance of cognitive errors in diagnosis and strategies to minimize them. Acad Med.

[7] Sbaraglia M, Bellan E, Dei Tos AP (2021). The 2020 WHO classification of soft tissue tumours: news and perspectives. Pathologica.

[8] Zell JA, Chang JC (2005). Neoplastic fever: a neglected paraneoplastic syndrome. Support Care Cancer.

[9] Kurzrock R (2001). The role of cytokines in cancer-related fatigue. Cancer.

[10] Hagi T, Nakamura T, Iino T, Matsubara T, Asanuma K, Matsumine A, Sudo A (2017). The diagnostic and prognostic value of interleukin-6 in patients with soft tissue sarcomas. Sci Rep.

[11] Nakamura K, Nakamura T, Iino T, Hagi T, Kita K, Asanuma K, Sudo A (2020). Expression of interleukin-6 and the interleukin-6 receptor predicts the clinical outcomes of patients with soft tissue sarcomas. Cancers (Basel).

[12] Wright WF, Auwaerter PG (2020). Fever and fever of unknown origin: review, recent advances, and lingering dogma. Open Forum Infect Dis.

[13] Gronchi A, Miah AB, Dei Tos AP (2021). Soft tissue and visceral sarcomas: ESMO-EURACAN-GENTURIS Clinical Practice Guidelines for diagnosis, treatment and follow-up☆. Ann Oncol.

